# Septischer Patient mit Fußgangrän: Ein Bericht über einen chirurgischen Notfall mit foudroyantem Verlauf

**DOI:** 10.1007/s00104-022-01674-z

**Published:** 2022-06-22

**Authors:** Mario Rastätter, Nike Walter, Susanne Bärtl, Volker Alt, Markus Rupp

**Affiliations:** Klinik und Poliklinik für Unfallchirurgie, Universitätsklinikum Regensburg, Franz-Josef-Strauß-Allee 11, 93053 Regensburg, Deutschland

## Anamnese

Ein 63-jähriger Patient wurde aufgrund seines sich zunehmend verschlechternden Allgemeinzustandes aus einem peripheren Krankenhaus in unsere interdisziplinäre Notaufnahme verlegt. Er stellte sich mit starken und seit vier Tagen bestehenden Schmerzen am linken Unterschenkel und Fuß vor. Er fühle sich zudem matt und angeschlagen, Fieber und Schüttelfrost wurden verneint. An relevanten Vorerkrankungen bestanden ein Typ 2b-Diabetes-mellitus mit diabetischer Polyneuropathie und peripherer diabetischer Angiopathie, Zustand nach Nicht-ST-Streckenhebungs-Myokardinfarkt (NSTEMI), periphere arterieller Verschlusskrankheit (PAVK) und eine arterielle Hypertonie.

## Befund

**Figure 1:**
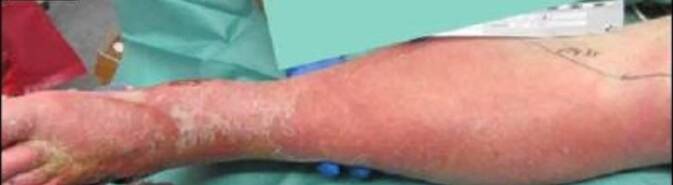

**Figure 2:**
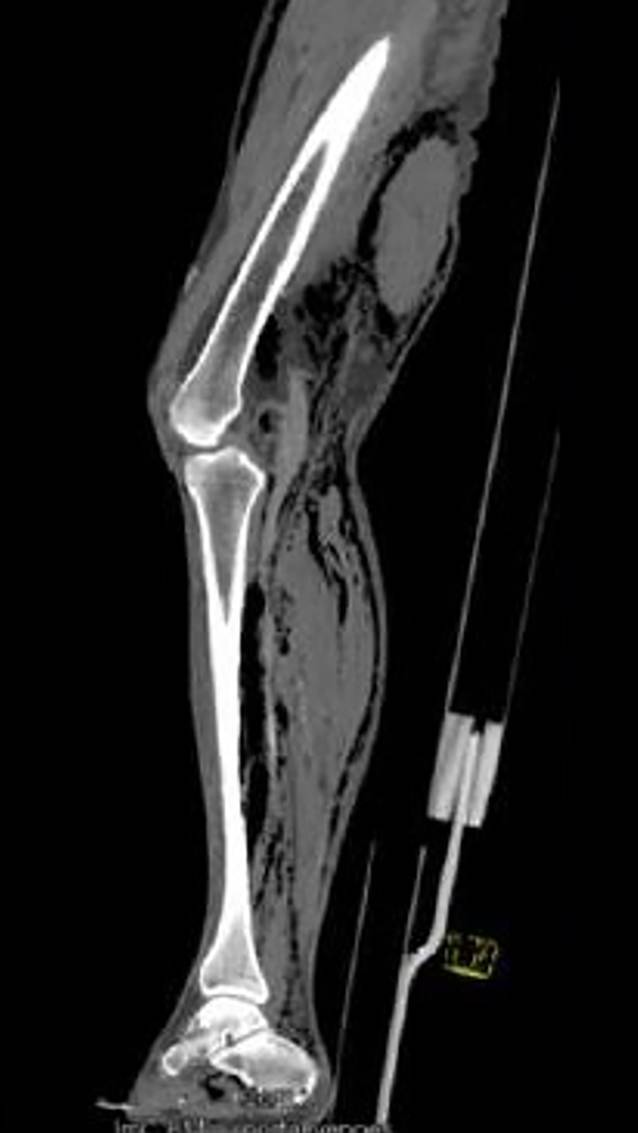

Der Patient war wach, wurde aber zunehmend somnolent und hatte einen reduzierten Allgemeinzustand und adipösen Ernährungszustand. Des Weiteren wurde eine Hypotonie (RR 80/50 mmHg) und eine Tachykardie (115/min) festgestellt. Es zeigte sich also bereits klinisch der Verdacht auf einen sich entwickelnden septischen Verlauf. Weiterhin fanden sich am ganzen Körper Petechien. Der Unterschenkel zeigte sich gerötet und geschwollen (Abb. 1). Außerdem fiel in der Palpation ein Knistern im Bereich der Wadenmuskulatur auf. Am linken Fuß fand sich eine feuchte Gangrän des fünften Zehs mit begleitender plantarbetonter Phlegmone. Laborchemisch fanden sich bei Aufnahme ein erniedrigtes Natrium (123 mmol/l), ein erhöhtes Kreatinin (5,5 mg/dl), ein erhöhtes C‑reaktives Protein (319 mg/l), erhöhte Leukozyten (20 × 10^9^/l) und ein erniedrigter Hämoglobinwert (11,1 g/dl). Die durchgeführte Computertomographie (CT) des Beins zeigte eine ausgedehnte Gasansammlung entlang der Faszien, die bis zur Hüftregion reichte (Abb. 2).

## Wie lautet Ihre Diagnose?

## Therapie und Verlauf

**Figure 3:**
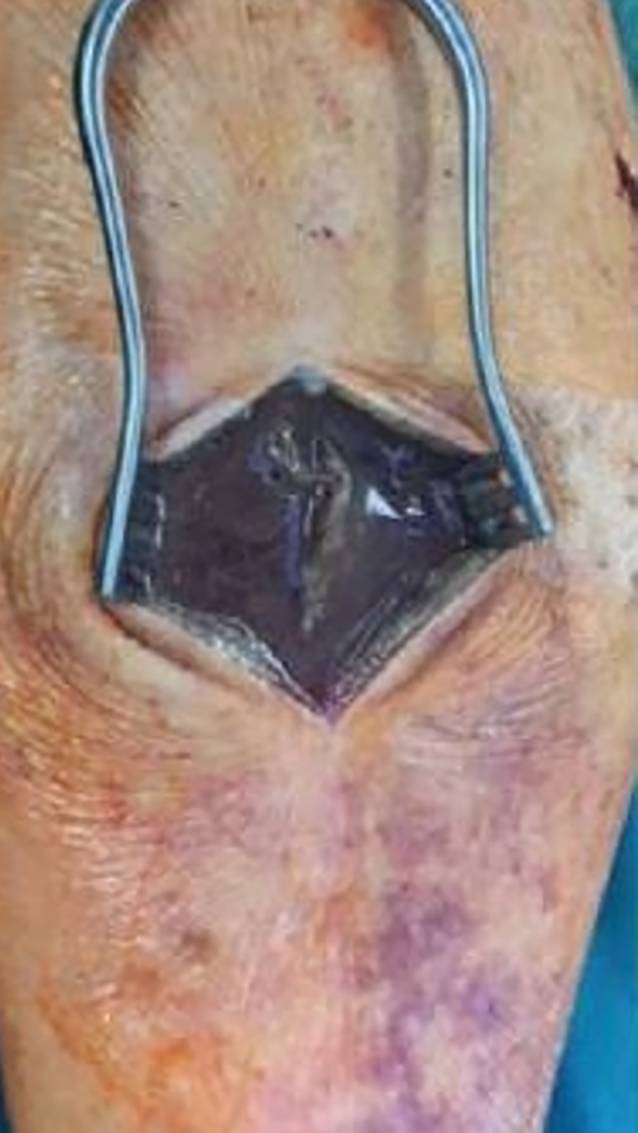

In Zusammenschau des klinischen, laborchemischen und CT-graphischen Bildes wurde die Diagnose einer nekrotisierenden Fasziitis gestellt und die Indikation zur notfallmäßigen, linksseitigen Oberschenkelamputation gestellt. Bereits bei Narkoseeinleitung musste der Patient aufgrund seines kritischen Zustandes reanimiert werden. Unterdessen wurde die Oberschenkelamputation links durchgeführt. Im Bereich des mittleren Oberschenkels zeigte sich kein Anhalt für eine bis hierauf reichende Fasziitis. Es konnten Granudacyn® (Mölnlycke, Düsseldorf, Deutschland) -getränkte Bauchtücher in den Situs eingelegt werden. Zusätzlich wurde auch eine Gewebeprobe aus dem Unterschenkel entnommen und zur pathologischen Untersuchung verschickt. Hier zeigte sich sowohl makroskopisch (Abb. 3) als auch mikroskopisch eine fortgeschrittene nekrotisierende Fasziitis. Nach der Operation wurde der Patient weiter auf der Intensivstation versorgt und erhielt weiter die bei Diagnosestellung begonnene vierfache Antibiotikatherapie mit Vancomycin, Meropenem, Penicillin G und Clindamycin.

Am nächsten Tag erfolgte eine Second-look-Operation. Intraoperativ zeigte sich eine überwiegend vitale Muskulatur mit einzelnen Nekrosen, die sorgfältig débridiert wurden. Auch in der aufsteigenden Hüftmuskulatur zeigte sich makroskopisch kein Anhalt für eine fortschreitende nekrotisierende Fasziitis. Aus dem Markraum des Femurs wurden mikrobiologische Proben entnommen und dieser anschließend mit Granudacyn® ausgespült. Nach Débridement der Muskulatur wurden auch in den Amputationsstumpf wieder Granudacyn®-getränkte Bauchtücher eingelegt. Die mikrobiologische Diagnostik ergab den Befund von *Staphylococcus aureus, Klebsiella oxytoca* und *Bacteroides fragilis*.

Postoperativ geriet der Patient auf der Intensivstation in ein ausgeprägtes Leber- und konsekutives Gerinnungsversagen und musste aufgrund einer starken Blutung aus der Operationswunde massentransfundiert werden. Aufgrund eines zwei Tage später aufgetretenen progredienten Multiorganversagens und erneuter volumenrefraktärer, hämodynamischer Instabilität und hohem Fieber wurde im Einvernehmen mit den Angehörigen und dem Behandlungsteam eine „Best-supportive-care“-Therapie eingeleitet. Der Patient verstarb noch am selben Tag.

## Definition und Diskussion

Die nekrotisierende Fasziitis ist eine rasch progrediente und potenziell lebensgefährlich verlaufende schwere Infektion des Weichteilgewebes, die sich entlang der Faszien ausbreiten und fulminante septische Verläufe induzieren kann. Sie wurde bereits im 5. Jahrhundert v. Chr. von Hippocrates beschrieben. Das US Centers for Disease Control Prevention (CDC) schätzt die jährliche Inzidenz auf ca. 0,4 Fälle pro 100.000 Einwohner [4]. Die Mortalität dieser Erkrankung wird in der Literatur mit etwa 20–30 % angegeben [2, 5].

Risikofaktoren sind neben hohem Alter und dem männlichen Geschlecht auch Diabetes mellitus, maligne Tumoren, Alkoholmissbrauch und chronische Leber- und Nierenerkrankungen. Die nekrotisierende Fasziitis präsentiert sich klinisch an der infizierten Stelle oft mit Erythem, Verhärtung, Schmerzempfindlichkeit und seltener mit Hautnekrosen und Bullae, wobei diese meistens im Bereich der Extremitäten, des Rumpfs oder des Perineums vorkommen. Letzteres stellt eine Sonderform dar und wird als Fournier Gangrän bezeichnet. Hinzu kommen noch vegetative Symptome wie Tachykardie, Hypotonie und Tachypnoe. Das Fehlen von Fieber ist auch durchaus häufig (44 %) und erschwert damit eine frühe Diagnosestellung [4].

Da die Schwierigkeit bei der nekrotisierenden Fasziitis sowohl in einer frühen Diagnosestellung als auch in der Abgrenzung zu einer nichtschwerwiegenden Weichteilinfektion besteht, wurde der LRINEC (Laboratory Risk Indicator for Necrotizing Fasciitis)-Score (Tab. 1) entwickelt, der nur anhand von Laborparametern das Risiko für eine nekrotisierende Fasziitis zu prognostizieren versucht. Dieser zeigt zwar in einer Metaanalyse eine positive Korrelation zur klinischen Diagnose, hat sich aber in der klinischen Praxis nicht durchgesetzt. Bildgebende Diagnostik in Form einer CT ist oftmals sinnvoll, um die Diagnose zu sichern. Ein Zeitverlust für die spätere Therapie sollte jedoch unbedingt vermieden werden. Bei unklarer Diagnose ist eine Probeinzision mit Biopsien für die Mikrobiologie und Pathologie zur Diagnostik zu entnehmen [1]. Oft findet sich auf der nekrotischen Faszie auch eine gräulich-trübe Flüssigkeit, das sog. „dishwater“. Das Vorliegen dieser stützt die Diagnose einer nekrotisierenden Fasziitis zusätzlich.

**Table 1:** 

Parameter	Wert	Scorepunkte
Hämoglobin (g/dl)	>13,5	0
	11–13,5	1
	< 11	2
Leukozyten (10^9^/l)	< 15	0
	15–25	1
	> 25	2
Natrium (mmol/l)	< 135	2
Kreatinin (mg/dl)	> 1,6 mg/dl	2
C‑reaktives Protein (mg/l)	> 150	4
Glukose (mmol/l)	> 10	1

^a^Der LRINEC(Laboratory Risk Indicator for Necrotizing Fasciitis)-Score ermöglicht eine Abschätzung des Risikos für eine nekrotisierende Fasziitis anhand der oben angegebenen sechs Laborparametern. Dabei gilt für den Score: ≤ 5 geringes Risiko, 6–7 mittleres Risiko, ≥ 8 hohes Risiko [1]

Die nekrotisierende Fasziitis lässt sich mikrobiologisch in vier Kategorien einteilen:

- Typ I stellt die häufigste Variante dar (55–90 %). Dabei handelt es sich um eine polymikrobielle Infektion, die auch bei dem hier vorgestellten Patienten vorlag. Besonders betroffen sind dabei Patienten mit Immunsuppression und Diabetes mellitus.
- Typ II bezeichnet eine Infektion mit Gruppe-A-Streptokokken (*Streptococcus pyogenes*).
- Typ III stellt eine Infektion mit *Clostridium *spp., gramnegativen Bakterien oder *Vibrio *spp. dar.
- Typ IV bezeichnet eine fungizide Infektion [2].

Bei der Therapie ist eine zügige Versorgung gemäß dem Motto „time is fascia“ [3] von eminenter Bedeutung. So zeigten Nawijn et al. in einer Metaanalyse, dass ein frühes chirurgisches Débridement, innerhalb der ersten 6 h, die Mortalität bei Patienten mit nekrotisierender Fasziitis um fast 50 % reduziert. Zudem wurde gezeigt, dass auch eine operative Versorgung innerhalb von 12 h eine Reduktion der Mortalität gegenüber der nach mehr als 12 h versorgten Gruppe herbeiführt [3].

Intraoperativ sollte der Fokus besonders darauf gerichtet werden, alle infizierten Gewebeanteile sowie zusätzlich den Rand der gesunden Faszie zu resezieren. Zusätzlich sollte auch immer initial eine antibiotische Therapie mit Ampicillin/Sulbactam und Clindamycin oder Metronidazol erfolgen [2].

Das hier präsentierte Gesamtbild aus einem LRINEC-Score von 10, die Gasansammlungen entlang der Faszie im CT, das Unterschenkelerythem, die Konstellation der Vitalparameter und der Risikofaktor Diabetes mellitus deuteten alle auf ein sehr hohes Risiko für eine nekrotisierende Fasziitis bei dem Patienten hin. So war bei diesem Fall aufgrund der genannten Befunde eine Diagnosestellung und operative Versorgung frühzeitig möglich.

**Diagnose:** nekrotisierende Fasziitis

Aufgrund der fulminanten septischen Entwicklung der nekrotisierenden Fasziitis, war es trotz evidenzbasierter Therapie jedoch nicht möglich, den Patienten zu retten.

## Fazit für die Praxis

- Als Ursache für einen septischen Verlauf sollte bei Weichteilinfektion eine nekrotisierende Fasziitis in Betracht zu gezogen werden.
- Die nekrotisierende Fasziitis ist eine rasch fortschreitende, lebensbedrohliche Erkrankung mit einer hohen Mortalität, die immer einen Notfall darstellt.
- Eine schnelle Diagnose- und Therapieeinleitung ist notwendig, um den schweren Krankheitsverlauf einer nekrotisierenden Fasziitis bestmöglich zu beeinflussen. Ein couragiertes Handeln ist hierfür unverzichtbar.
